# Experimental and numerical study on the shear performance of stainless steel-GFRP connectors for use in precast concrete sandwich panels

**DOI:** 10.1038/s41598-024-64543-1

**Published:** 2024-06-15

**Authors:** Boyi Zhao, Lingfeng Du, Guixiang Chen, Longfei Yue, Chenxing Cui, Mengmeng Ge

**Affiliations:** 1 Civil Engineering School, Henan University of Technology, Zhengzhou, China; 2 School of Civil and Transportation Engineering, Henan University Of Urban Construction, Pingdingshan, China

**Keywords:** Precast concrete sandwich panel, Connectors, Shear capacity, Structural testing, Numerical analysis, Theoretical analysis, Engineering, Civil engineering

## Abstract

Precast Concrete Sandwich Panel (PCSP) is composed of concrete load-bearing panels, thermal insulation panels, and decorative panels, which are assembled through connectors, integrating load-bearing, thermal insulation, and decorative functions. The connector bears the main shear force between the wall panels, and the shear resistance and insulation performance of the connector largely determine the mechanical stability and insulation effect of the wall panels, which is a key component in PCSPs. The current common practice is to cross assemble stainless steel insulation (SSI) connectors and Glass-Fiber-Reinforced Plastic (GFRP) connectors into PCSPs, which can reduce building energy consumption and save resources while meeting strength and insulation requirements. A large-scale pull-out test on a PCSP with intersecting SSI-GFRP connectors was conducted in this paper. The damage process and damage pattern of PCSP were observed and the shear performance of SSI-GFRP connectors was analyzed. Secondly, a numerical analysis model of the test PCSP was built using ABAQUS finite element software and its validity was verified through the test data. In addition, parameters such as connector diameter, connector number ratio and concrete strength were analyzed for their effect on the shear performance of SSI-GFRP connectors and it was found that connector diameter and connector number ratio had a significant effect. Finally, it is found that there are some differences between the classical theory for calculating the shear performance of SSI-GFRP connectors and the actual results. A theoretical correction factor (*ζ*) is given to improve the accuracy of the calculation of the classical theory, and its influencing factors and changing rules are investigated.

## Introduction

Precast concrete sandwich panels (PCSPs), commonly utilized components in assembled buildings, consist of external concrete wall and load-bearing interior concrete wall, along with thermal insulation panels, interconnected via connectors, offering excellent thermal insulation alongside primary load-bearing functions^1–4^. PCSP integrates heat preservation, load-bearing, and partitioning, serving as an environmentally friendly, low-carbon, energy-efficient building component aligned with the principles of green construction and low-carbon environmental stewardship^5,6^. Connectors, bearing the maximum shear force between wall panels, are pivotal components of PCSPs, and their performance significantly influences the overall effectiveness of the PCSP. Currently, research concerning the shear resistance and insulation capabilities of PCSPs primarily centers on investigating the material properties, stress distribution, and layout configurations of connectors.

Commonly utilized connector materials include metals (e.g., stainless steel) and non-metallic materials (e.g., fiber-reinforced materials)^7,8^. Metal connectors offer excellent shear resistance, fire resistance, ductility, and affordability^9^. Metal connectors are a viable option when corrosion resistance and wall insulation performance are not primary concerns^10,11^. Bush and Stine^12^ investigated truss-type metal shear connectors in PCSPs, demonstrating their ability to offer strong shear resistance and enhance the composite bending performance of wall panels. Goudarzi et al.^13^ examined the out-of-plane bending performance of composite wall panels utilizing novel steel plate connectors, with a focus on evaluating the influence of width and thickness on the shear strength and stiffness of these connectors. Lou et al.^10^ evaluated the shear behavior of stainless steel plate connectors in PCSP through shear tests, considering the effects of shear direction, cavity width and connector height, and the results showed that stainless steel plate connectors have better in-plane shear performance. Kinnane et al.^14^ conducted shear performance tests on 25 precast sandwich panels equipped with steel plate shear connectors to analyze the effects of different cavity widths and connector sizes on the overall structural shear capacity, and the results showed that steel plate connectors have excellent mechanical properties. The above studies show that the good shear resistance of metal connectors ensures the safety and stability of the structure. However, the high thermal conductivity of the metal connectors themselves often leads to obvious thermal bridging effects^15,16^, which seriously affects the thermal insulation performance of the wall panels. Naito et al.^17^ observed through experimental research that the shear resistance of steel truss connectors surpasses that of FRP connectors, emphasizing the need for caution regarding heat and corrosion resistance considerations. Kim and Allard^18^ studied in detail the thermal performance of PCS walls with W-type, Z-type and J-type steel connectors and found that W-type steel connectors have lower thermal conductivity. Yu et al.^19^ found that metal connectors of different forms and lengths differed in the thermal performance of the wall, but their thermal conductivity was greater than that of GFRP connectors. The poor thermal performance of metal connectors should not be ignored, which is a prominent problem.

To address thermal bridge issues, fiber-reinforced polymers have become the primary choice in engineering for replacing steel in the manufacturing of new connectors. Typical non-metallic materials comprise Glass-Fiber-Reinforced-Plastic (GFRP), Basalt-Fiber-Reinforced-Plastic (BFRP), Carbon-Fiber-Reinforced-Plastic (CFRP), and Aramid-Fiber-Reinforced-Plastic (AFRP)^20–22^. GFRP stands out as the commonly utilized material for connectors, extensively employed in engineering applications. GFRP exhibits relatively low thermal conductivity, thereby enhancing the insulation performance of PCSPs. Woltman et al. conducted tests and simulations to evaluate the thermal resistance of a PCSP featuring a novel GFRP shear connector, observing a reduction in thermal bridge effects compared to steel connectors^23^. Sylaj et al. devised and examined a double-layer insulation wall panel incorporating ultra-high performance concrete (UHPC) and GFRP connectors, resulting in reduced weight and enhanced insulation performance of the panel^24^. Nevertheless, GFRP connectors exhibit poor mechanical properties, making them susceptible to shear failure, and their durability is significantly influenced by environmental factors^25^. Huang et al. extensively examined the shear resistance and failure modes of various types of GFRP connectors, finding that longitudinal shear failure and fiber tensile failure were prevalent among them^26^. Choi et al. conducted double-shear rollout experiments on PCSP wall panels equipped with grid-type GFRP connectors and found that the shear flow capacity tended to decrease as the thickness of the insulation layer increased^27^. Woltman et al. found that the shear strength of GFRP connectors is significantly higher than polymer connectors but lower than steel connectors^28^. Xue et al. found that the tensile strength and elastic modulus of GFRP connectors gradually decreased with rising temperature and prolonged exposure time in alkaline environments^29^. Owing to the reduced interlayer shear strength of GFRP connectors, they exhibit a higher susceptibility to shear failure at the perforated web^30,31^. In summary, fiber connectors offer advantages in pull-out resistance and thermal insulation, albeit with drawbacks such as low strength, low ductility, limited temperature resistance, and poor corrosion resistance. Conversely, stainless steel connectors excel in shear strength, thermal insulation, fire resistance, durability, and corrosion resistance, albeit with slightly higher thermal conductivity and cost. Thus, the predominant approach at this stage involves utilizing a combination of stainless steel and fiber connectors in a cross configuration within PCSPs, leveraging their respective strengths in strength and thermal insulation to mitigate individual weaknesses and meet project requirements effectively.

However, further elucidation is required regarding the operational performance of Stainless steel-GFRP connectors for implementation in PCSPs. To address this, an exploratory study was conducted on a novel type of insulated wall panels incorporating both stainless steel insulation (SSI) and GFRP connectors. A full-scale push out test was performed on the wall panels, and the finite element method was employed to analyze the impact of factors such as quantity ratio, connector diameter and concrete strength on the shear performance of the wall panel connectors. This analysis provides preliminary insights into the integrated shear performance of SSI-GFRP connectors for use in PCSPs and elucidates the load–displacement change rule.

## Materials and methods

### Description of test specimens and materials

**Figure 1:**
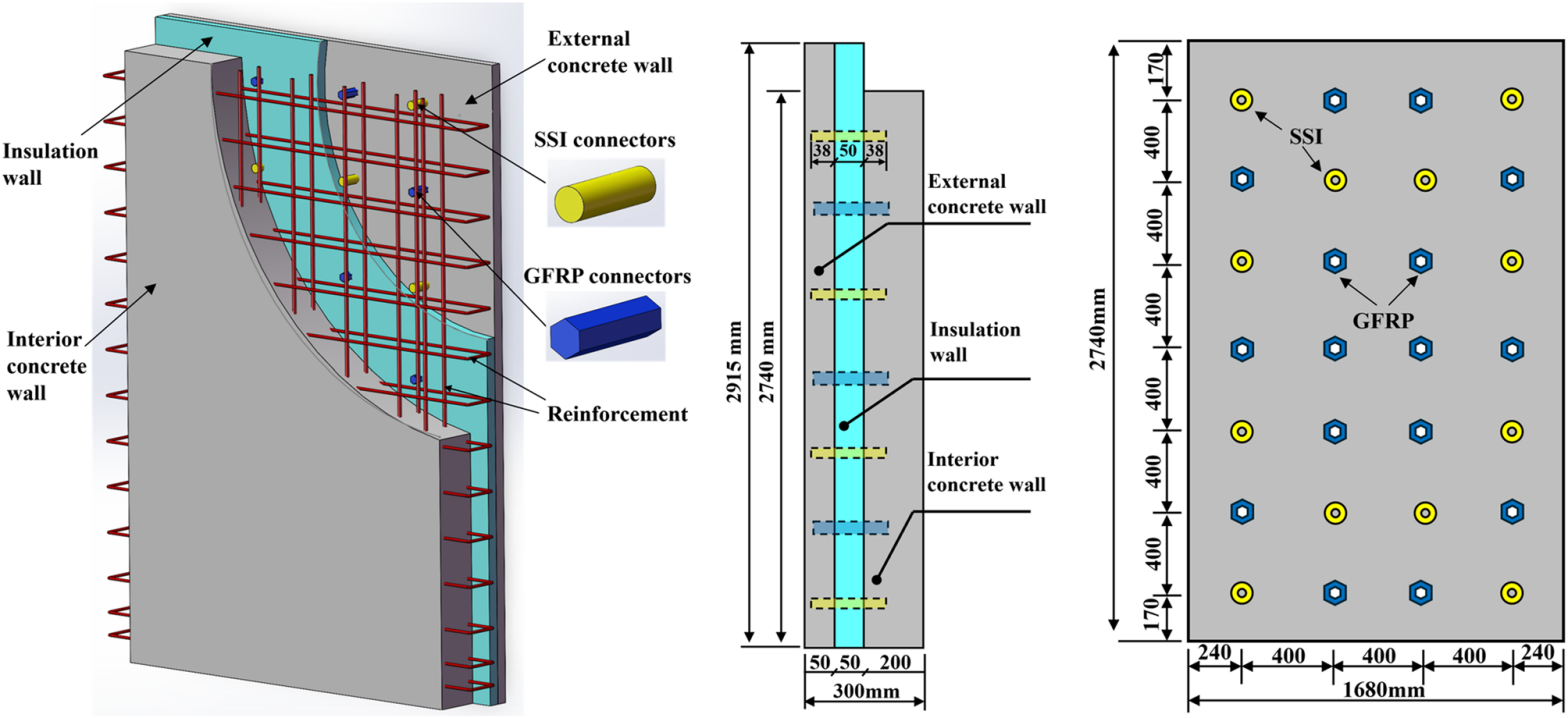
Construction and schematic diagram of PCSP.

A new precast concrete sandwich panel measuring 1790 × 2915 mm (70.47 × 114.76 inches) has been fabricated, featuring a total wall panel thickness of 300 mm (11.81 inches) (Fig. 1). The panel consists of a 200 mm (7.87 inches) interior wall panel, a 50 mm (1.97 inches) insulation board, and a 50 mm (1.97 inches) external wall panel. Notably, the interior wall panel made of C35 concrete with a double layer of steel mesh, as load-bearing structures, is mainly responsible for supporting the vertical loads. Simultaneously, the insulation board acts as a barrier, ensuring thorough insulation of the wall panel. It is constructed from polystyrene material, commonly referred to as XPS board. The external wall panel made of C35 concrete with a single layer of steel mesh is a thin layer used mainly for decorative purposes. Furthermore, the connectors within the wall panel predominantly consist of two types: Stainless steel insulation (SSI) connectors and Glass-Fiber-Reinforced Plastic (GFRP) connectors, primarily tasked with bearing the shear force between each panel. Detailed material performance parameters of the connectors and steel are provided in Table 1.

**Table 1:** Material properties of connectors and rebars.

Type	Diameter (mm)	Density (g/cm^3^)	Tensile strength (MPa)	Elastic modulus (MPa)	Poisson ratio
GFRP	10	2.0	316.8	4.47 × 10^4^	0.2
SSI	12	7.9	530	2 × 10^5^	0.3
Rebar	8/10/12	7.9	400	2 × 10^5^	0.3

### Experimental procedure

**Figure 2:**
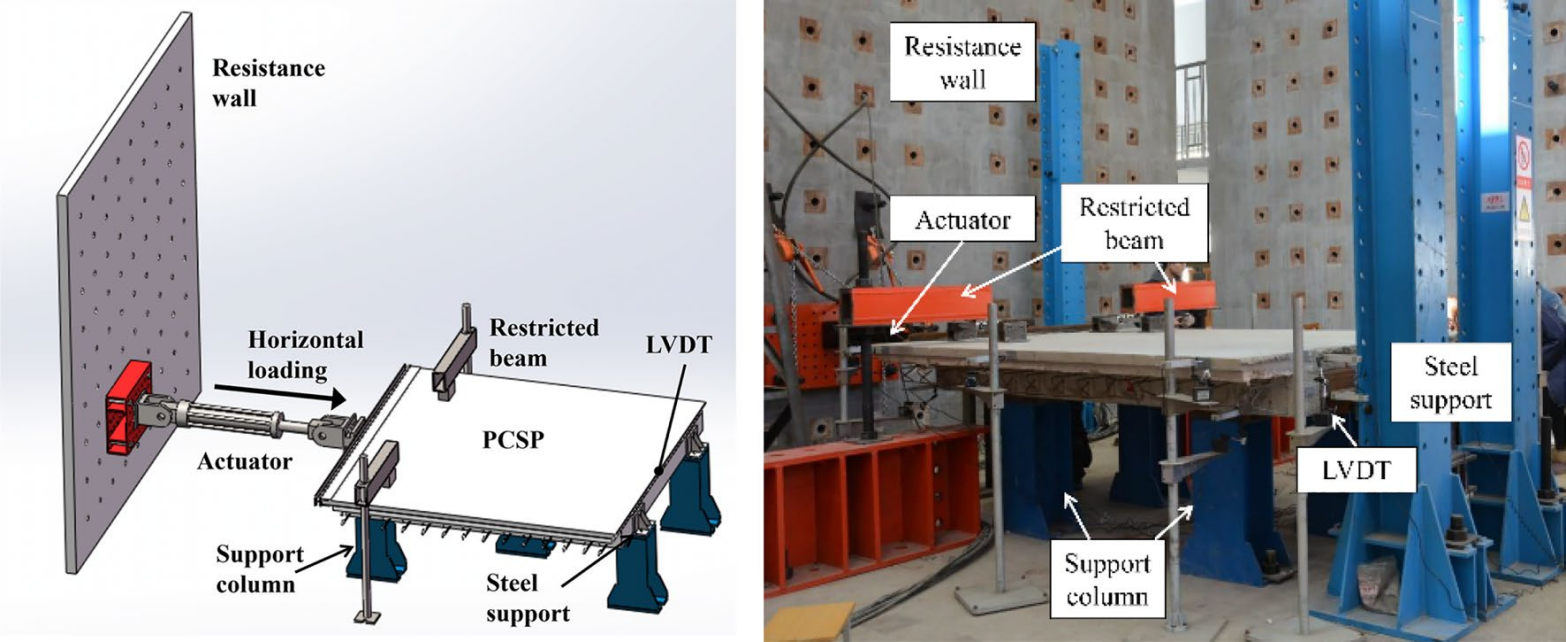
Schematic diagram and photos of test device.

After 14 days of natural curing, the wall undergoes horizontal monotonic static loading. As depicted in Fig. 2, the specimen is initially elevated using four short support columns anchored on the ground. The height of these four short columns is meticulously regulated by the testers using hydraulic pressure to ensure the specimen remains on the same horizontal plane. Subsequently, a bull leg device is positioned on one of the wall panels (located on the right in the figure) to restrict the horizontal displacement of the interior wall panels. The remaining four short support columns beneath the specimen and the two compression beams positioned above it (as shown in Fig. 2) are equipped with sliding devices to minimize interference with the specimen's horizontal movement. Finally, on the opposite side of the specimen, a horizontal stress actuator is arranged to apply horizontal thrust to the center of the external concrete wall, which is anchored to the reinforced concrete reaction wall. The actuator is secured to a steel plate with large screws and aligns flush with the proximal end of the plate. Displacement gauges are positioned at the center of the distal end of the external concrete wall to measure the bottom displacement of the external concrete wall.

**Figure 3:**
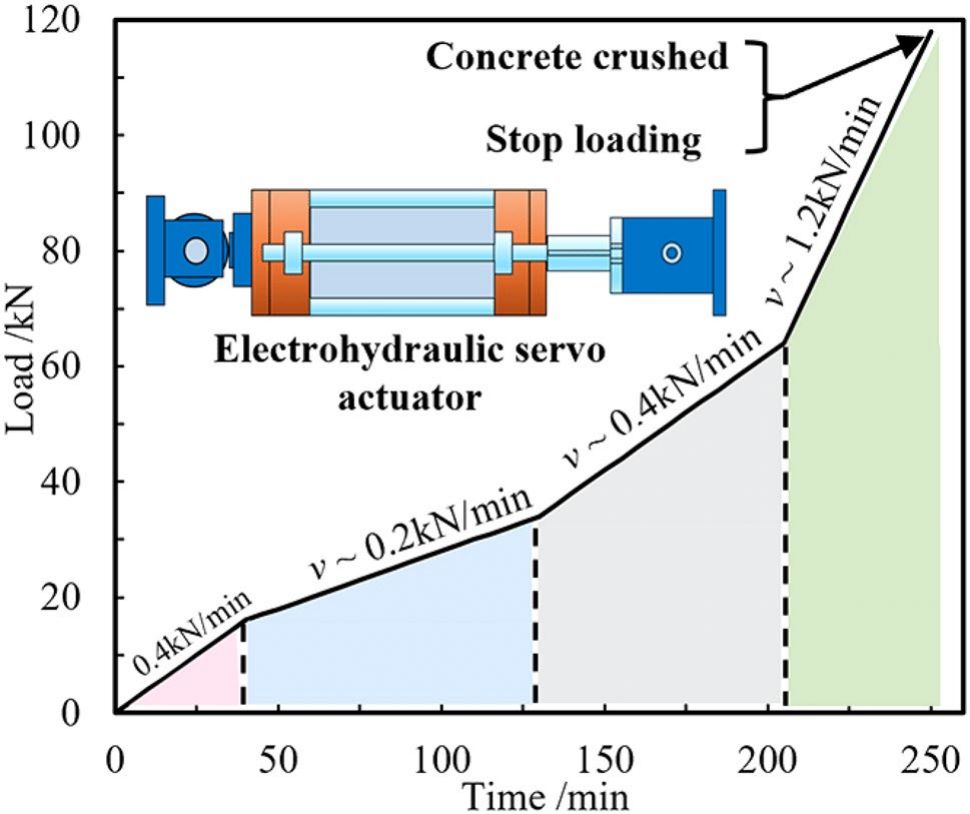
Test loading steps.

Before loading, apply 1–3 levels of preload and monitor real-time data to observe whether the specimen, devices, and instruments are functioning normally and to troubleshoot any issues. After ensuring that the experimental devices and acquisition equipment are working properly, remove the preload, zero the load, and commence the formal loading stage. Horizontal graded loads (0.4 kN/min, 0.2 kN/min, 0.4 kN/min, 1.2 kN/min) are applied at reduced rates using the end actuator to ensure more stable displacement data can be measured at each level of loading. The specimen loading procedure is illustrated in Fig. 3.

## Experimental results and analysis

### Failure modes

Throughout the test, the external concrete wall of the PCSP experienced horizontal thrust from the actuator, causing horizontal displacement in the in-plane direction. The horizontal thrust exerted on the external concrete wall is transmitted through the axially arranged connectors, thereby subjecting the connectors themselves to shear and tensile effects. Moreover, the shear and tensile effects on the connectors propagate to the thermal insulation board and the interior wall panel, leading to localized stress concentration phenomena in both components. Besides the externally applied loads, friction occurs between the plates, and bond-slip occurs between the connectors and the plates. These different stresses interact and superimpose to cause various damage patterns in PCSP.

**Figure 4:**
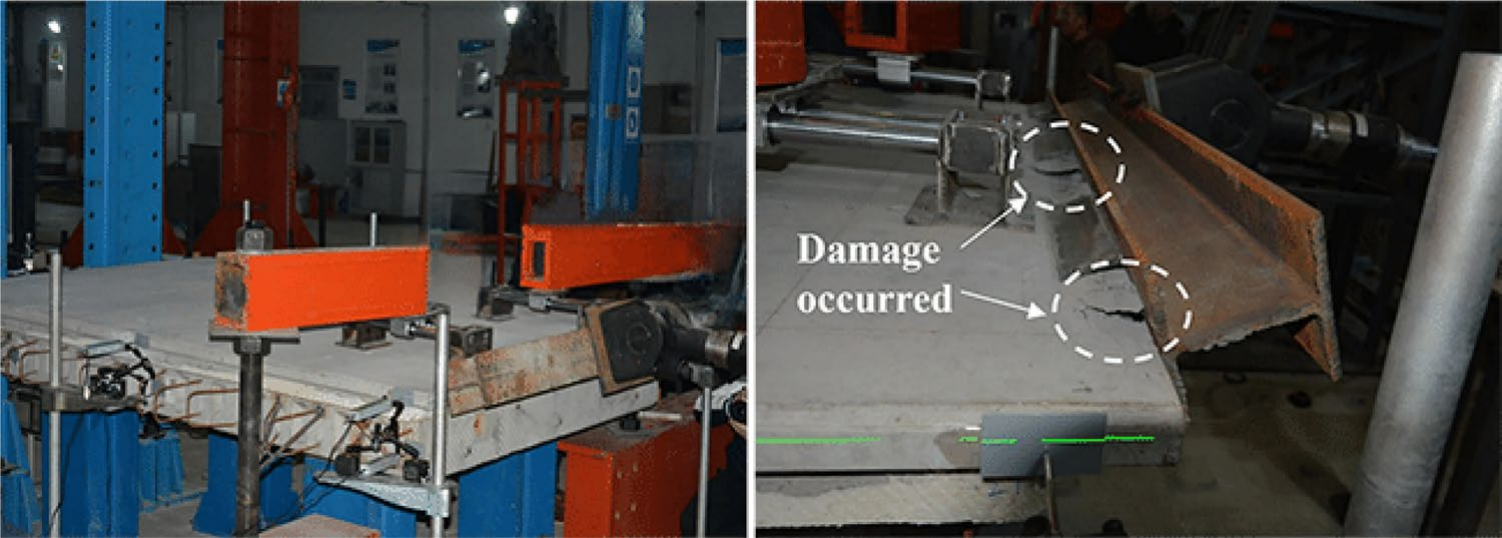
Photo of damaged specimen.

As the horizontal thrust applied by the actuator gradually increases, the displacement of the external panel also gradually increases, and the various types of connectors undergo synchronized minor slips and bending deformations. The connector ends bears the bending moment when subjected to shear force. Transfer of the bending moment to the concrete layer results in the preferential appearance of horizontal cracks on the inside of the concrete external concrete wall in contact with the joint, leading to tensile damage to the concrete. With further increase in load, the residual bearing capacity decreased gradually and damage to the concrete increased. At the same time, horizontal cracks were observed in the interior wall panels, and minor warping occurred in the external concrete wall. Upon loading the specimen to 118 kN, the external wall panel of the PCSP experienced localized crushing damage at the end due to the combined effects of horizontal thrust and bending moment, thereby concluding the test. Figure 4 shows photos of the destruction.

### Load–displacement curves

**Figure 5:**
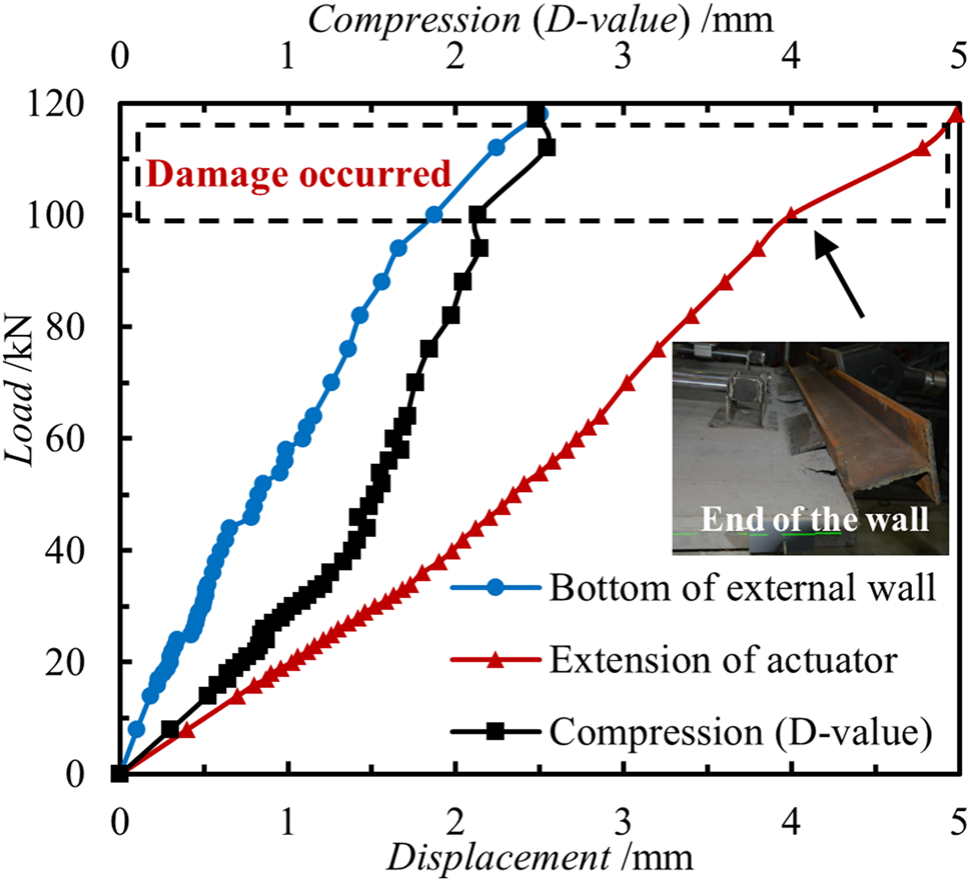
Load–displacement curves of test.

Figure 5 displays the load–displacement curves of the specimen. The two curves correspond to the displacement at the actuator (equivalent to the proximal center) and the displacement at the distal center of external concrete wall (equivalent to the distal center, as depicted in Fig. 2), respectively.

Upon analysis of the load–displacement curves in the figure, the stress deformation of the external concrete wall of the PCSP can be categorized into 3 stages. Initially, within the range of 0 to 30 kN, the displacement increases linearly with the load. At this juncture, the stress level is low, and the member undergoes elastic deformation, which comprises two components: elastic compression of the concrete and elastic deflection deformation of the joints. Subsequently, when the load reaches 30 kN, the rate of displacement change exhibits an inflection point, after which the rate decreases until 100 kN. At this stage, in addition to the elastic deformation, the shear force and bending moment on the connectors further increase, and the connectors starts to deform plastically, while localized damage and cracking occurs in the concrete slab where the connectors is anchored. Finally, when the load reaches 100 kN, the displacement of the moving device rapidly increases, while the displacement of the distal center remains stagnant. The cracks in the concrete wall panels widened further, accompanied by greater tensile damage. Upon observation of damage to the end parts of the actuator (such as concrete cracking or local crushing), the experiment is terminated, as depicted in Fig. 4.

## Numerical and theoretical analysis

The nonlinear analysis of the PCSP was performed using the Finite Element Analysis (FEA) package, ABAQUS/Standard module. The validity of the model was verified using experimental results, and a series of parametric analyses were performed to study the shear performance of SSI-GFRP connectors. In addition, based on the results of finite element analysis, a theoretical correction factor is given to optimize the theoretical calculation results.

### Material constitutive model

**Figure 6:**
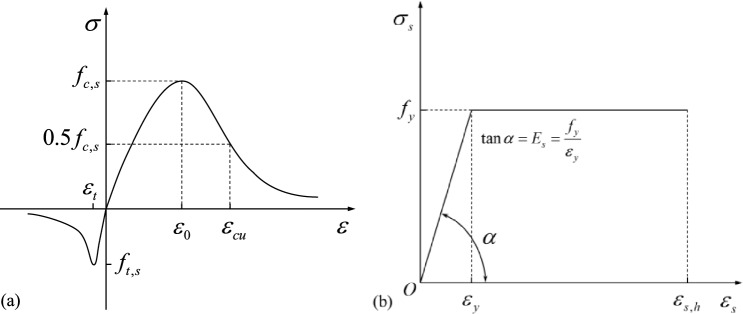
Stress–strain curves for concrete and rebar.

In this experiment, the interior concrete wall and the external concrete wall of the PCSP are modeled using the concrete plastic damage model. The stress–strain curve is depicted in Fig. 6, and the concrete material properties are summarized in Table 2. The Concrete Damaged Plasticity (CDP) model is well-suited for simulating unidirectional loading, repetitive loading, and dynamic loading of brittle materials, and exhibits good convergence^32^. The model is grounded in the theory of isotropic elastic damage and plasticity in tension or compression, enabling simulation of concrete's inelastic behavior such as tension cracking and compression crushing. It considers stiffness degradation due to plastic strain through the damage factor in tension and compression^33^. Additionally, based on the stress–strain relationship curves of rebar under unidirectional loading, the bifold model (Fig. 6) was chosen for simulation in this study.

**Table 2:** Concrete property parameters.

Density	Modulus of elasticity	Poisson's ratio	Dilation angle	Centrifugal rate	*f*_*b0*_/*f*_*c0*_	*K*	Viscosity coefficient
2.5 g/cm^3^	31,027	0.2	36.31	0.1	1.16	0.667	0.005

GFRP connectors are constructed from fiberglass material. According to Deng et al.^34^, fiberglass material exhibits linear elastic behavior without a significant yield point. The GFRP connectors in this study were subjected to a single direction of force, and the softening stage and damage deformation after the connectors reached the limit state were not considered, which is assumed to be a linear elastic material in this study. The primary material used for SSI connectors is 304 steel. The stress–strain curves of GFRP and SSI connectors are similar to Fig. 6b. The key material property parameters of the reinforcement utilized for numerical analysis are presented in Table 1. The insulation material in the sandwich wall panels consists of extruded polystyrene (XPS), which experienced minimal or no loading during the tests. To simplify the analysis, the insulation material in the model is treated as an ideal elastic material.

### Establishment and verification of finite element model

**Figure 7:**
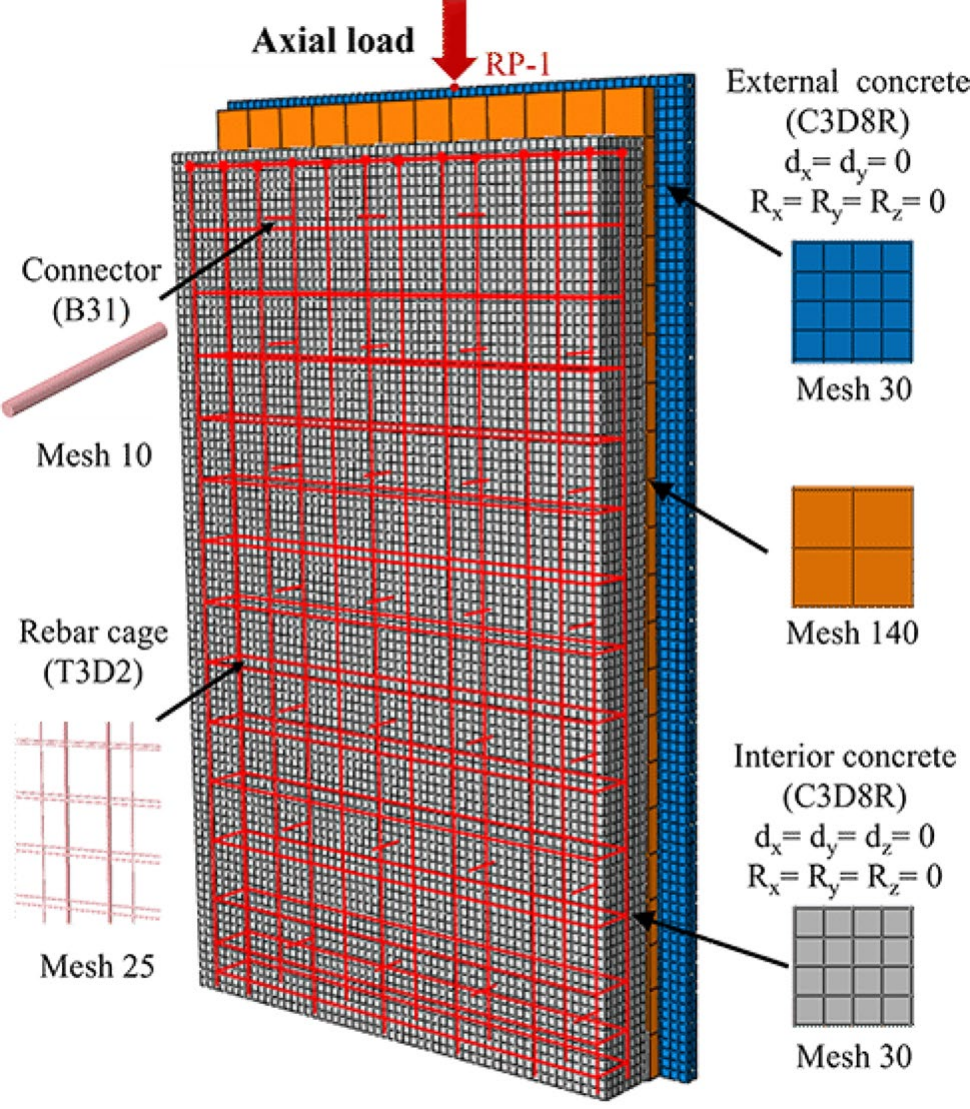
Schematic of finite element model and mesh.

For the numerical simulation of the shear strength of sandwich wall panel connectors, the selected component types for each unit are as follows: C3D8R solid units are chosen for concrete and insulation board, T3D2 truss units are selected for steel reinforcement, and B31 beam units are utilized for connectors. Each part of the model mainly contains two kinds of contact methods, which are mainly defined using *Surface to Surface* and *Embedded Region* of Interaction function module in ABAQUS. The steel cage and steel mesh are embedded in the interior concrete wall and external concrete wall respectively. The connectors are embedded into three walls simultaneously. The relationship between the surface of the insulation wall and concrete wall is defined in terms of *Surface* *to* *Surface* contact. Normal behavior of both adopted “hard” contact, whereas the tangential behavior adopted the “penalty” friction formulation with a friction coefficient of 0.3^28,35^. In order to balance computational accuracy and efficiency, the grid size is set to 15 for rebars and connectors and 30 for concrete wall panels. The schematic of the model and the meshing diagram are presented in Fig. 7.

Boundary conditions are applied to the model, mirroring those of the experimental setup, whereby the bottom surface of the interior wall panels is fully constrained from displacement. Reference point RP-1 is established and coupled with the top surface of the external concrete wall to facilitate load application across the entire surface. Utilizing the displacement loading mode, a fixed downward displacement of 4 mm is applied to reference point RP-1, with simultaneous output of the bearing reaction force and displacement during the calculation. Furthermore, reference point RP-2 is positioned at the center of the floor of the external concrete wall to monitor the displacement of the external concrete wall throughout the calculation process. The iterative analysis of the ABAQUS software is terminated when the bottom displacement of the external concrete wall reaches 4 mm. At this point, the calculation process of numerical simulation is considered completed, and the data of loads, displacements, stresses, and damages during the analysis process are collected for in-depth analysis.

**Figure 8:**
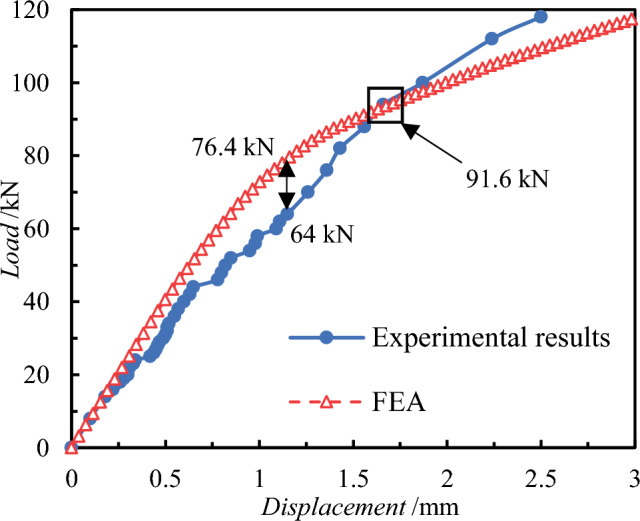
Load–displacement curves for tests and FEA.

**Figure 9:**
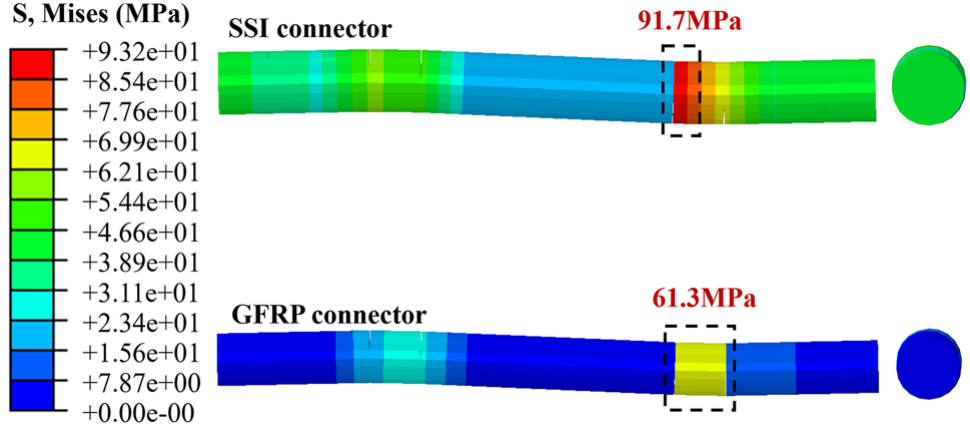
Mises of SSI and GFRP connectors.

**Figure 10:**
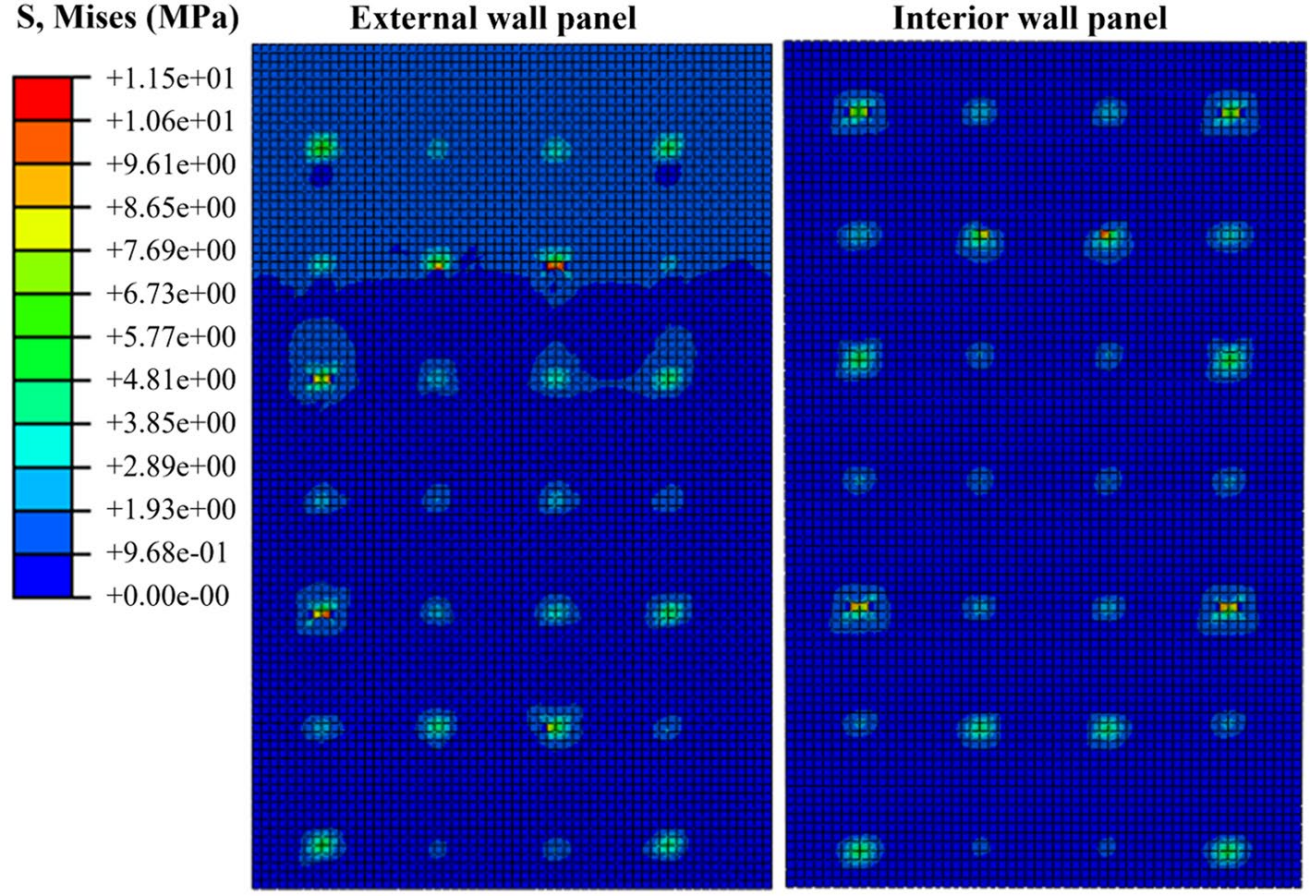
Mises of External concrete wall and Interior concrete wall.

**Figure 11:**
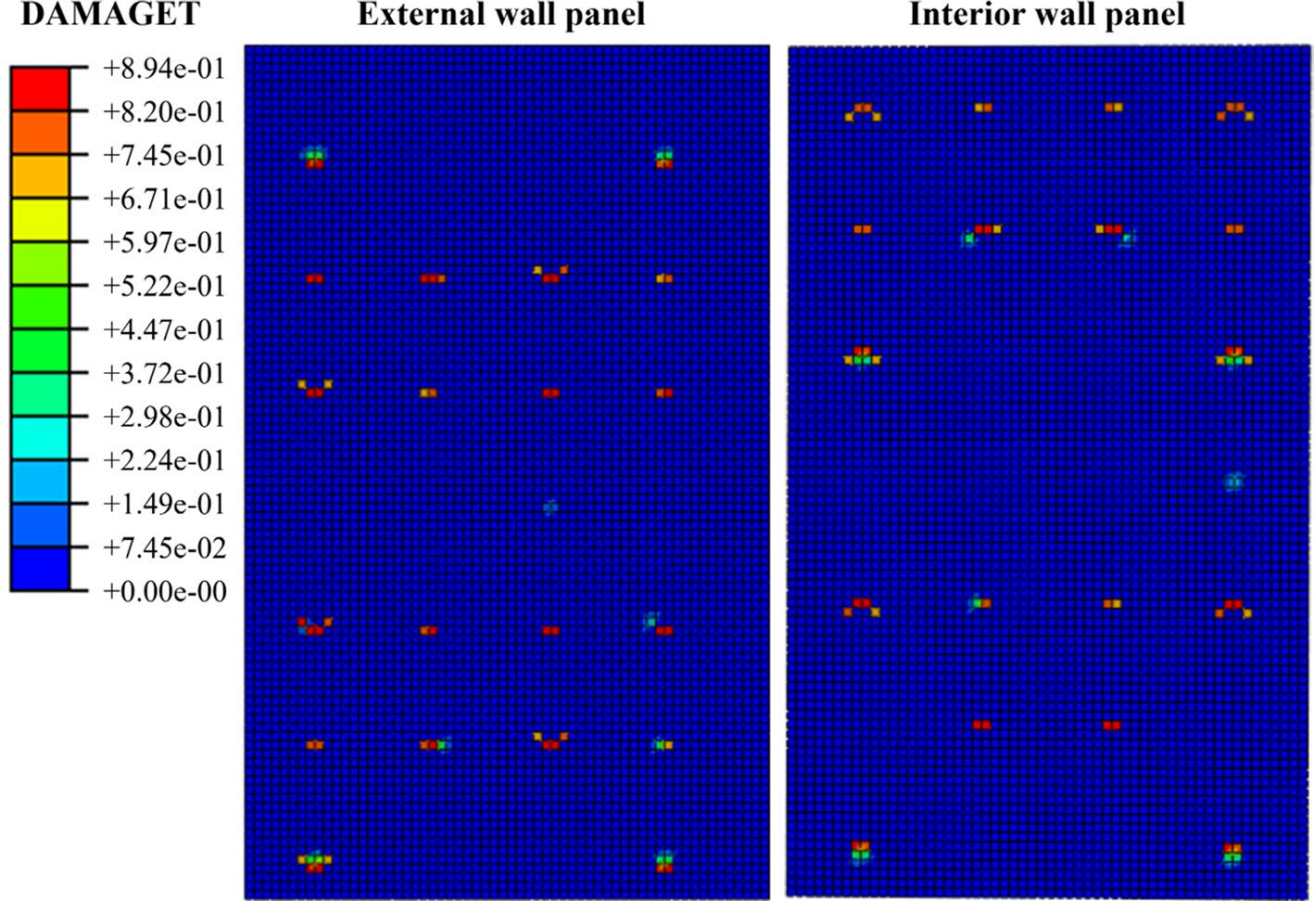
Concrete tensile damage of External concrete wall and Interior concrete wall.

The finite element calculation results reveal the load–displacement curves of the external concrete wall in Fig. 8, while the stress and damage results post-load extraction are depicted in Figs. 9, 10, 11. Initially, the stress diagram of concrete wall panels reveals that the stress generated due to the bending deformation of the connectors is transferred to the interior and external wall panels, with stress concentration occurring primarily at the contact position between the connectors and the two wall panels post-loading. Additionally, greater stress is observed where the wall panels are constructed with SSI connectors, suggesting that the SSI connectors endure larger shear stresses during loading.

Importantly, Figure 11 shows that the damage of the concrete interior and exterior wall is mainly concentrated at the anchoring position of the connectors, which is consistent with the phenomenon of concrete crushing around the connectors in the experiment. Additionally, a comparison of the load–displacement curves between the test and the finite element calculation reveals an 85% similarity between the two results. This analysis demonstrates the successful verification of the finite element calculation model established in this section.

### Parametric analysis

#### Types of connectors

The experimental wall panel consists of 28 connectors, comprising 16 GFRP connectors and 12 SSI connectors, arranged in a uniform staggered configuration. To assess the influence of various ratios of connector quantities on the comprehensive shear performance of SSI-GFRP connectors in PCSP, this section developed six PCSP numerical models with proportions identical to those of the test wall panel, while maintaining a constant total number of connectors. The difference among the models is solely in the number and proportion of different connectors, with detailed analysis conditions provided in Table 3. The normal service load of components in Table 3 refer to the actual loads on the external concrete wall at the end of the numerical simulation analysis.

**Table 3:** Analysis of working conditions for different quantities of SSI and GFRP connectors.

Component number	GFRP	SSI	The ratio of SSI/GFRP	Normal service load of components (kN)
WB-Test	16	12	0.75	130.84
WB1	28	0	0	100.03
WB2	20	8	0.4	118.63
WB3	14	14	1	137.08
WB4	12	16	1.33	143.02
WB5	8	20	2.5	155.10
WB6	0	28	–	178.63

**Figure 12:**
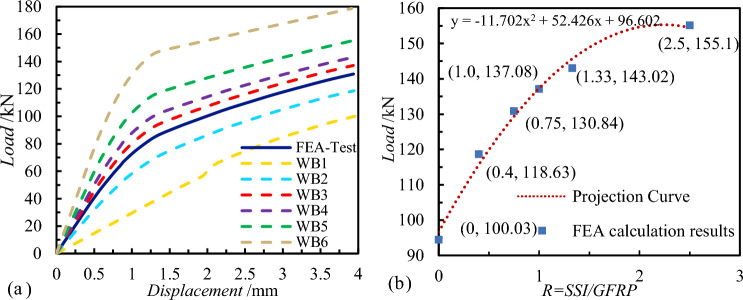
Numerical calculation results: (**a**) Load–displacement curves; (**b**) The relationship between load and connector quantity ratio.

Analysis of the load displacement curves reveals significant disparities among individual curves, suggesting that varying numbers and ratios of connectors exert a substantial impact on shear performance. In Fig. 12a, across WB1-WB6, the normal operating loads of the wall panels range from 100.03 to 178.63 kN. Generally, the normal service load of wall panels increases with the number of SSI connectors, indicating a gradual improvement in the comprehensive shear performance of SSI-GGRP connectors. SSI connectors, due to their higher shear stiffness, play a major role in shear performance, similar to the research findings of previous scholars. The normal service loads of components, along with the *R* (the quantity ratios of SSI and GFRP), were graphed in the same coordinate system to examine the relationship between loads and connector ratios, depicted in Fig. 12b. The shear capacity of the wall panel connectors increases with the ratio of SSI to GFRP. When *R* is gradually increased with each case, the percentage of final load lifting over the previous condition is 18.6%, 10.3%, 4.8%, 4.3%, 8.4%, and 15.2%, respectively. However, the curve in Fig. 12b exhibits a gradually decreasing slope, signifying that the rate of increase in shear capacity of the wall panel connectors diminishes as the *R* increases. Although SSI connectors make a significant contribution to the shear performance of connectors, there is an optimal ratio between the two types. Thus, configuring a high proportion of SSI connectors to maximize shear capacity of wall panels is scientifically unsound and undesirable. Additionally, while SSI connectors offer high shear capacity, their thermal bridging effects should not be disregarded.

**Figure 13:**
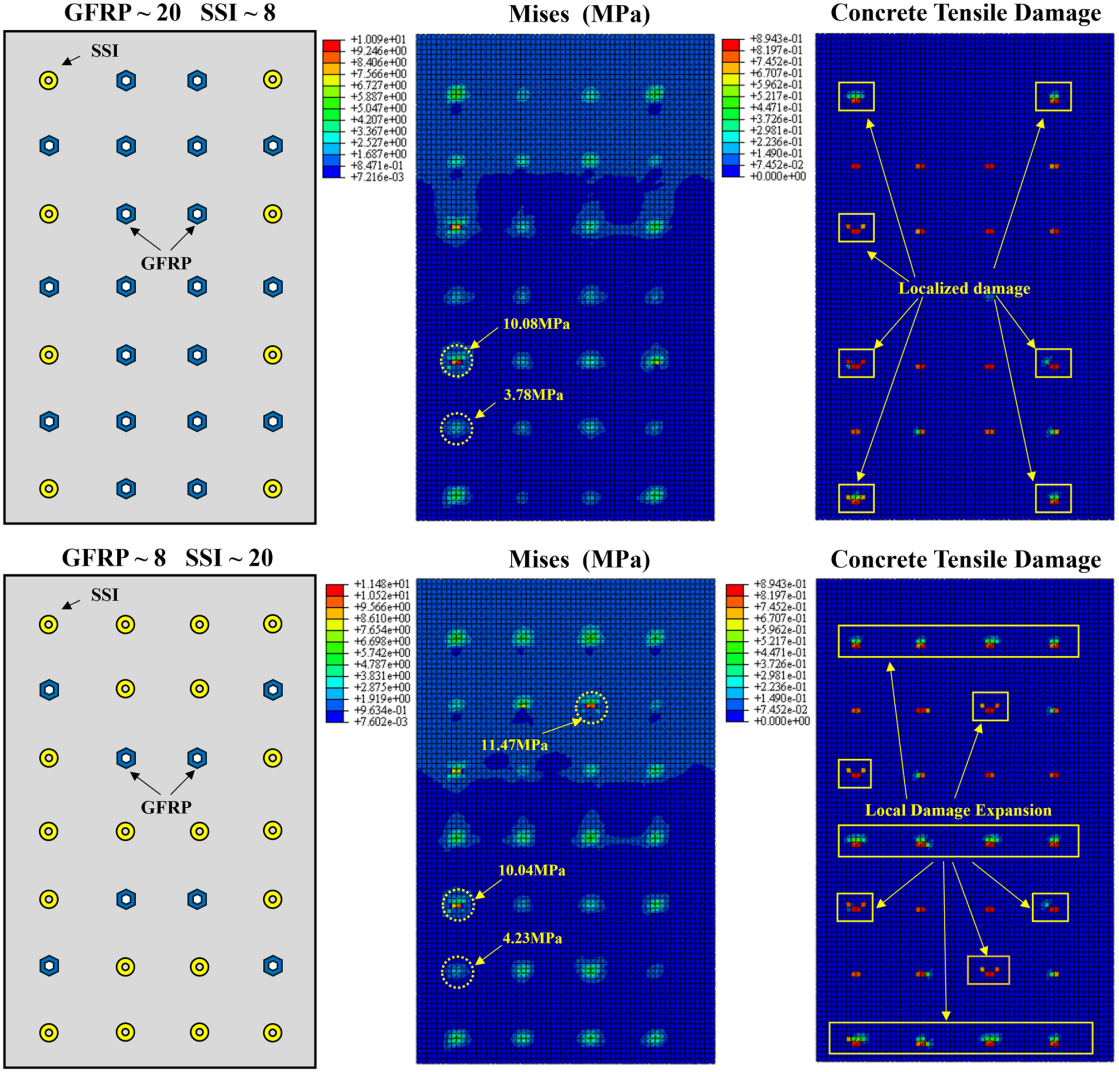
Mises and concrete tensile damage of the external concrete wall.

During the process of horizontal push out of the external concrete wall, all connectors play a role in resisting shear force, but the shear resistance provided by different connectors varies due to their own properties. Figure 13 shows the stress and concrete tensile damage results of the external concrete walls of WB2 and WB5. The stress diagram of the external concrete wall panel shows that there is a concentration of stress at the anchoring position of the connectors, and there is an uneven distribution. In the stress diagram of WB2, the stress value of the wall panel at the anchorage SSI is 10.08 MPa, while the stress value of the wall panel at the anchorage GFRP is 3.78 MPa. Stress concentration is more pronounced where SSI connectors are positioned, exhibiting larger stress extremes. Similarly, the damage to the concrete of the external concrete wall follows a pattern (Fig. 13), with noticeable damage occurring at the locations of anchored SSI connectors, while locations with anchored GFRP connectors exhibit minimal or no damage. It can be visualized that with the increase in the number of SSI connectors, the number of plastic damage points above the concrete slab increases and the extent of plastic damage expands. Moreover, the locations with severe concrete damage happened to be anchored with SSI connectors. The above phenomenon indicates that the increase in the number of SSI connectors leads to a gradual increase in the degree of localized tensile damage to the concrete, which further increases the unevenness of the wall panel damage and the risk of wall panel instability.

#### Diameter of connectors

The test wall panels feature two connector sizes: 10 mm diameter GFRP connectors and 12 mm diameter SSI connectors. To assess the impact of connector diameter variation on the overall shear performance of the sandwich wall panel connectors, six numerical models were created in ABAQUS software. The sole distinction among the models lies in the diameter of the connectors, with detailed analysis conditions provided in Table 4.

**Table 4:** Analysis of working conditions for different diameter of connectors.

Component number	GFRP	SSI	Cross-sectional area of the connectors	Normal service load of components (kN)
WB-Test	10	12	191.54	130.84
WB6	10	8	153.86	91.93
WB7	10	10	157	107.08
WB8	10	14	232.36	163.95
WB9	8	12	163.28	116.98
WB10	12	12	226.08	151.05
WB11	14	12	266.9	178.76

**Figure 14:**
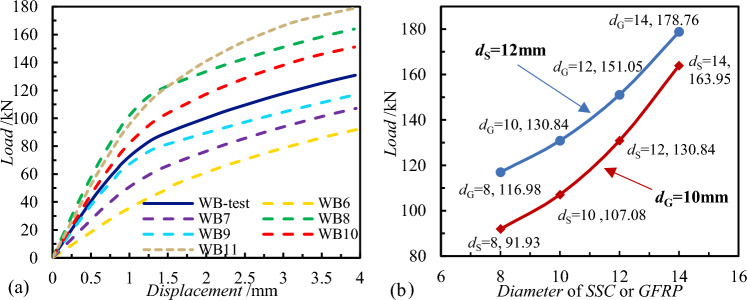
Numerical calculation results with different connector diameters.

**Figure 15:**
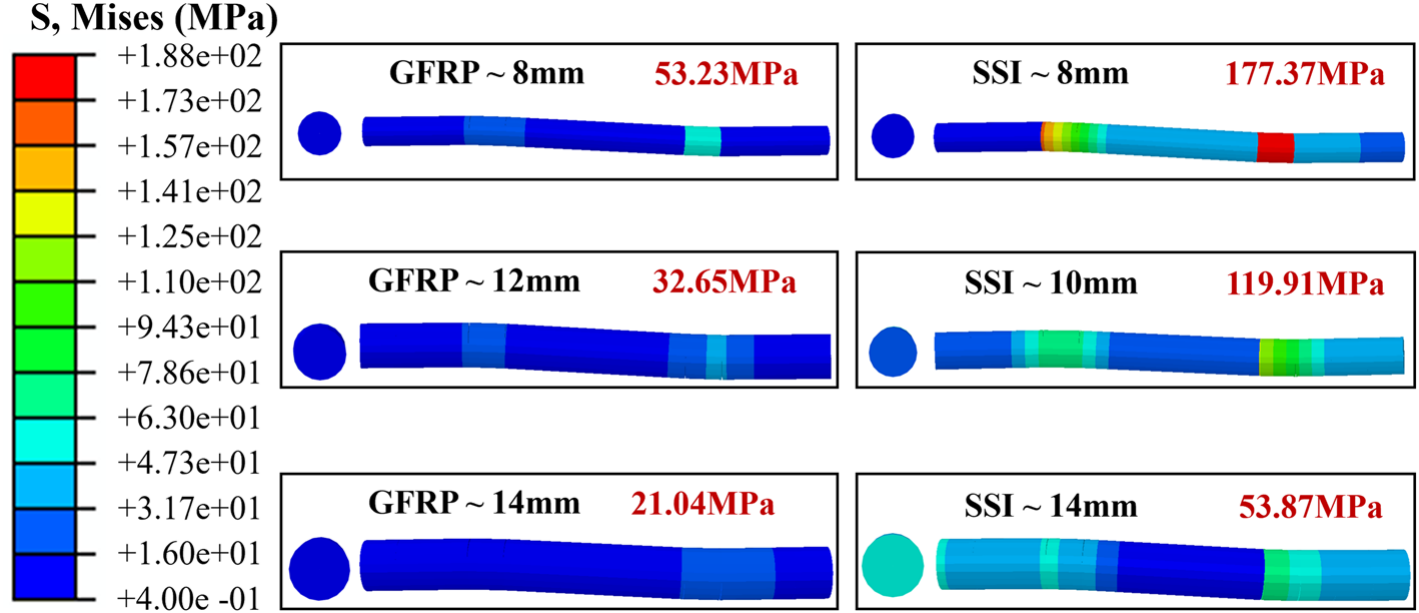
Stress diagrams for connectors of different diameters.

The numerical calculation results are shown in Fig. 14. Initially, the load–displacement curve in Fig. 14a is examined, revealing a gradual increase in the normal service load of the wall panel with increasing connector diameter, signifying a corresponding rise in shear capacity. Maintaining a 10 mm diameter for the GFRP connector, the normal service load of the wall panel varies as follows with different diameters for the SSI connector: 91.93 kN, 130.84 kN, 107.08 kN, and 163.95 kN for diameters of 8 mm, 10 mm, 12 mm, and 14 mm, respectively. With the diameter of the SSI connector set at 12 mm, the normal service load of the wall panel varies as follows with different diameters for the GFRP connector: 116.98 kN, 130.84 kN, 151.05 kN, and 178.76 kN for diameters of 8 mm, 10 mm, 12 mm, and 14 mm, respectively. It was found that when the diameter of the connectors was increased in incremental steps of 2 mm, the contribution of the two connectors to the enhancement of the shear capacity of SSI-GFRP was different. Subsequently, the shear performance of wall panel connectors was quantitatively analyzed by manipulating the diameter of GFRP or SSI connectors. As shown in Fig. 15, the stress of a single connector gradually decreases with increasing diameter while maintaining the same displacement load, indicating that the ability of the connector to be strained by the external load decreases, reflecting that the common shear capacity of the SSI-GFRP connectors improves. Particularly noteworthy is that, when maintaining the GFRP connector constant, the stress exhibits greater variability with increasing diameter of the SSI connector. At a diameter of 8 mm for the SSI connector, the stress measures 177.37 MPa; however, with an increase in SSI diameter to 12 mm and 14 mm, the stress diminishes to 119.91 MPa and 53.87 MPa, respectively.

**Figure 16:**
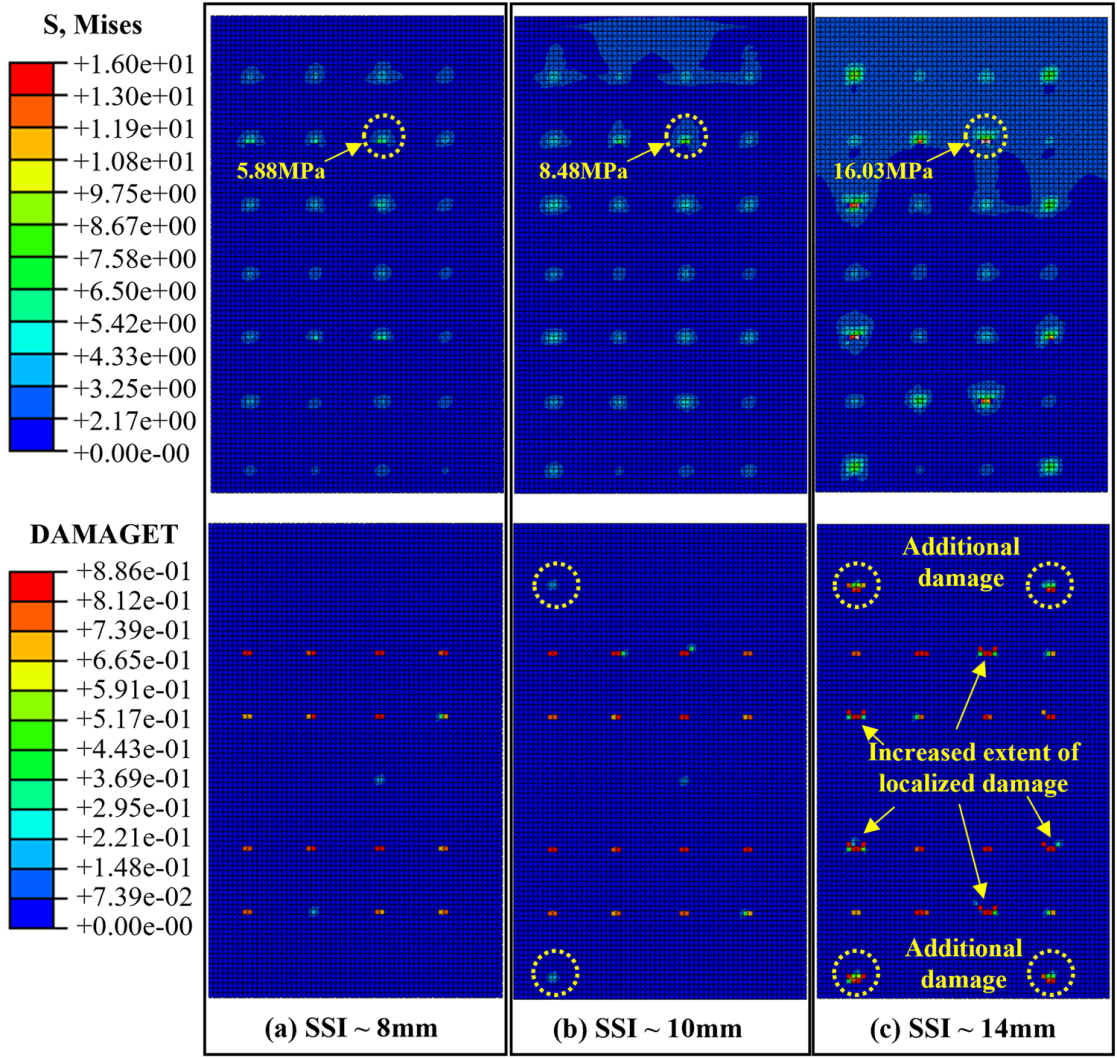
Mises and concrete tensile damage of external walls with different SSI diameters.

As the diameter of the connector increased, the stress concentration phenomenon and damage at the anchorage end of the external concrete wall became more pronounced. Illustrated in Fig. 16, maintaining the diameter of GFRP connectors constant while adjusting the diameter of SSI connectors from 8 to 14 mm results in a more uneven stress distribution within the concrete of the external concrete wall: stress shifts from a previously multi-point distribution to primarily concentrate at the position of SSI connectors, with the maximum stress increasing from 5.88 to 16.03 MPa. Simultaneously, the damage to the external concrete wall is exacerbated, with more localized damage points and an enlarged local damage range.

#### Concrete of different strengths

**Figure 17:**
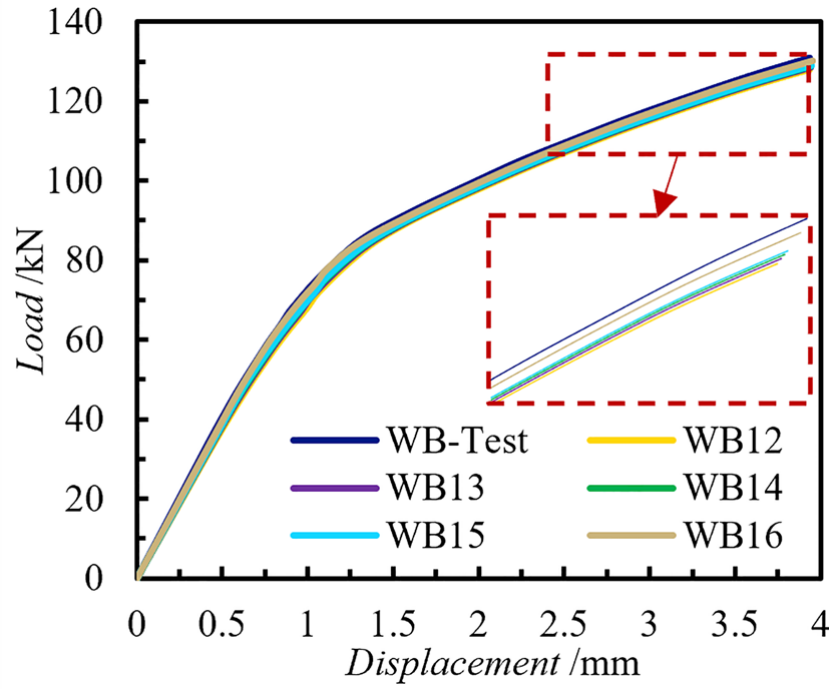
Normal service load of PCSPs with different concrete strengths.

Five numerical calculation models were established, based on the numerical validation tests, to explore the impact of concrete strength grade on the integrated shear performance of connectors in PCSPs. The models differ solely in concrete grade, ranging from C30 to C60 for the interior and external wall panels of the sandwich wall panels. Additionally, ultra-high-performance concrete (UHPC) is utilized in this section as a computational case to examine the shear performance of sandwich wall panel connectors configured with UHPC. The analysis of working conditions for different concrete strengths are shown in Table 5. Upon analyzing the load–displacement curves depicted in Fig. 17, it was observed that the results of each numerical calculation model were consistent, suggesting that the concrete strength grade has minimal impact on the shear performance of the connectors in PCSPs.

**Table 5:** Analysis of working conditions for different concrete strengths.

Component number	Concrete Grade	Compressive strength of concrete (MPa)	Normal service load of components (kN)
WB-Test	C35	35	130.84
WB12	C30	30	128.06
WB13	C40	40	128.41
WB14	C50	50	128.67
WB15	C60	60	128.92
WB16	UHPC	120	130.04

**Figure 18:**
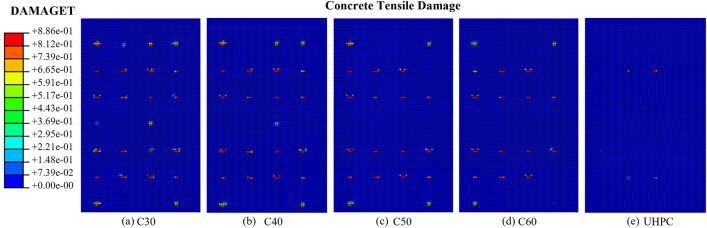
Tensile damage contours of external concrete walls.

Analysis of the stress and damage contours of the external concrete wall reveals a decrease in stress concentration with higher concrete strength grades, resulting in fewer local damage points and reduced damage values (Fig. 18). As the concrete grade increased, the extent of tensile damage to the concrete was significantly reduced and the rate of damage propagation was significantly reduced, although no change in bearing capacity could be observed in the load–displacement curves. The ability of the PCSPs to resist damage and cracking was improved.

### Theoretical analysis

**Figure 19:**
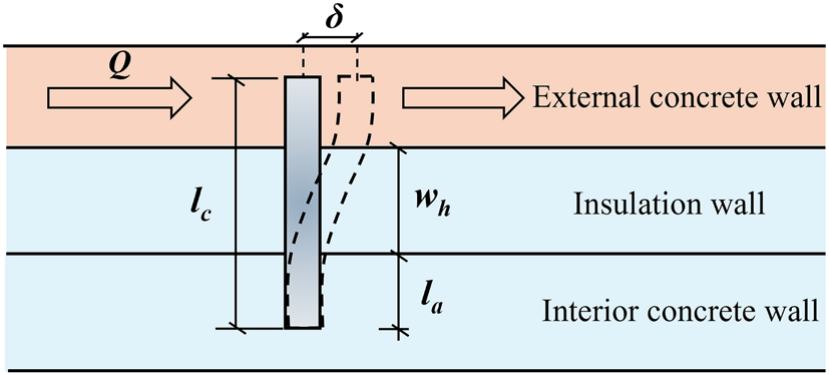
Calculation diagram of connectors.

The effects of different factors on the shear performance of SSI-GFRP connectors are generally clarified through the experimental and numerical analyses in the previous section. In this section, the shear performance of SSI-GFRP connectors of the PCSP in this study will be verified and analyzed by theoretical analysis. According to the ICC AC320 criterion^36^, the displacement of the connectors in the PCSP caused by gravity load can be calculated by Eq. (1), and the calculation sketch is shown in Fig. 19. Prior to the theoretical analysis, the following basic assumptions were specified: (1) the compressive behavior of the external wall panel itself during the stressing process was ignored; (2) the effect of bond-slip of the connectors with the sandwich composite wall panel was not considered; and (3) the incremental displacement occurring in the connectors by the load was assumed to be equal to that occurring at the bottom of the external wall panel slab.

1 $$\delta = \frac{{Q \cdot l_{c} }}{{12E_{c} \cdot I_{c} }}$$

where, *δ* is the displacement caused by the load, *Q* is the load averaged over the individual connectors, *E*_*c*_ is the modulus of elasticity of the connectors, *I*_*c*_ is the moment of inertia of the connectors (*I*_*c*_ = πd^4^/64), and *l*_*c*_ is the computed length of the connectors, which can be computed by using Eq. (2).

2 $$l_{c} = w_{h} + \frac{{2l_{a} }}{3} \cdot \left( {1 - \frac{1}{{1 + l_{a} /w_{h} }}} \right)$$

where *w*_*h*_ is the thickness of the insulation board of the PCSP and *l*_*a*_ is the embedment depth of the connectors in the concrete. The above theoretical approach was used to calculate the shear performance of the SSI-GFRP connectors in the PCSP. By applying the same displacements as in the previous numerical analysis, the actual theoretical load that should be applied were back-calculated to study the differences between the theoretical and numerical analysis values. The calculation formula is derived as Eq. (3).

3 $$Q = m\frac{{12E_{cS} \cdot I_{cS} \cdot \delta }}{{l_{c} }} + n\frac{{12E_{cG} \cdot I_{cG} \cdot \delta }}{{l_{c} }}$$

where *m* and *n* are the number of SSI and GFRP connectors, respectively, and *E*_*cS*_, *I*_*cS*_, *E*_*cG*_, *I*_*cG*_ are the modulus of elasticity and moment of inertia corresponding to SSI and GFRP, respectively.

**Figure 20:**
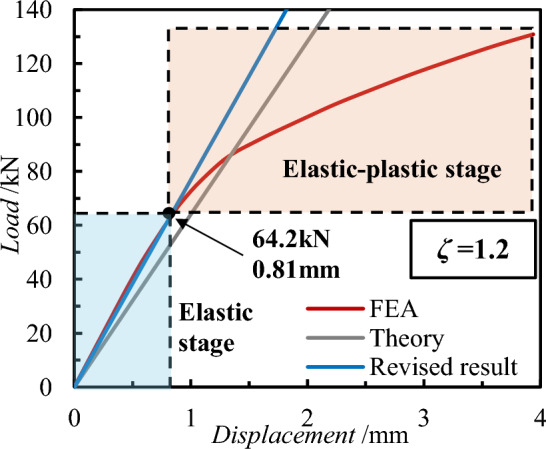
Comparison of results between theoretical analysis and FEA.

Using Eq. (3), the theoretical results of loads and displacements of the test PCSP connectors were calculated and compared with the numerical analyses, as shown in Fig. 20. As shown in the figure, the results of finite element analysis exist elastic and elastic–plastic stages. The theory of deformation of connectors subjected to loads is only for the elastic stage, and the results of its computational analysis present a linear relationship. This study focuses on the analytical results of SSI-GFRP connectors in the elastic phase. We observe that there is a partial difference between the theoretical results and the finite element results in the elastic phase. Considering this phenomenon, this study concludes that the classical theory may have limitations that lead to differences between the predicted analytical results and the actual results, which may affect the practical application of the theory. Therefore, in order to optimize and correct the analytical results of the classical theory, a theoretical correction factor *ζ* is given in this study to optimize the further application of the classical theory (Eq. (4)).

4 $$Q = \xi \cdot \left( {m\frac{{12E_{cS} \cdot I_{cS} \cdot \delta }}{{l_{c} }} + n\frac{{12E_{cG} \cdot I_{cG} \cdot \delta }}{{l_{c} }}} \right)$$

For the shear performance of SSI-GFRP connectors in this study, when *ζ* is equal to 1.2, the results of the theoretical analysis can be fitted well with the elastic stage of the finite element analysis results, which proves the validity and accuracy of the correction factor. When the displacement value reaches 0.81 mm and the load value reaches 64.2 kN, the finite element analysis results start to appear plasticity, indicating the end of the elastic section. In order to further study the variation range and law of the correction coefficient *ζ*, the different parameters of the working conditions of the finite element were checked through the above theory.

**Figure 21:**
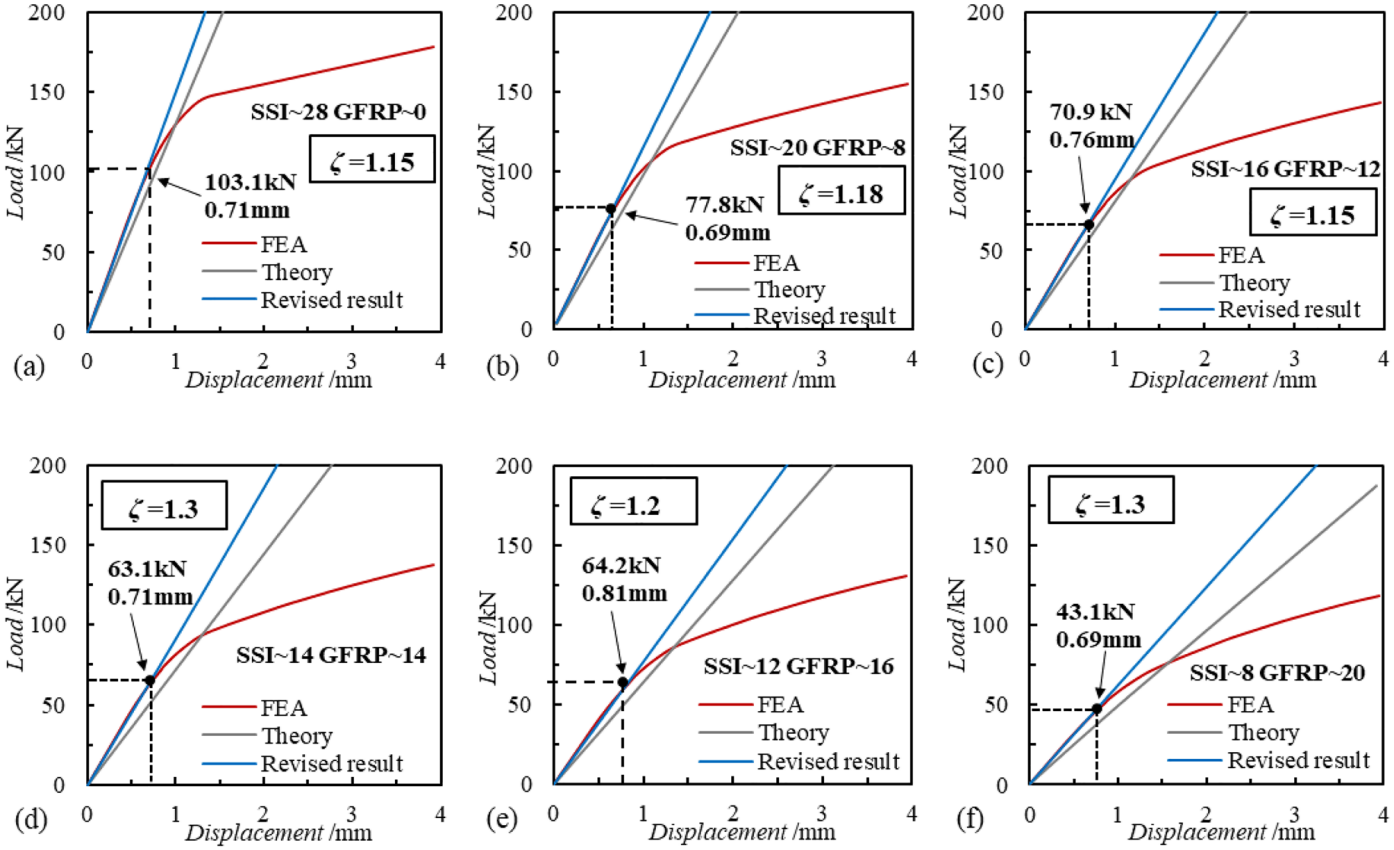
Theoretical analysis with different ratios of SSI and GFRP connectors.

Firstly, Fig. 21 demonstrates the results of the theoretical analysis under conditions with different ratios of the number of SSI and GFRP connectors. Overall, the theoretically calculated load–displacement curves all differ from the load–displacement curves of the finite element analysis, which need to be optimized. With the increase of the number of SSI connectors, the elastic section of the shear working performance of SSI-GFRP connectors ended at 103.1 kN, 77.8 kN, 70.9 kN, 63.1 kN, 64.2 kN, and 43.1 kN, respectively, which indicated that the elastic stage was getting shorter and shorter. According to the theoretical calculation results of different number of connectors ratio working conditions, different theoretical correction coefficients are given to make the theoretical results more close to the finite element analysis results. The calculation results show that the correction coefficient is in the range of 1.15–1.3, and the average value is 1.21.

**Figure 22:**
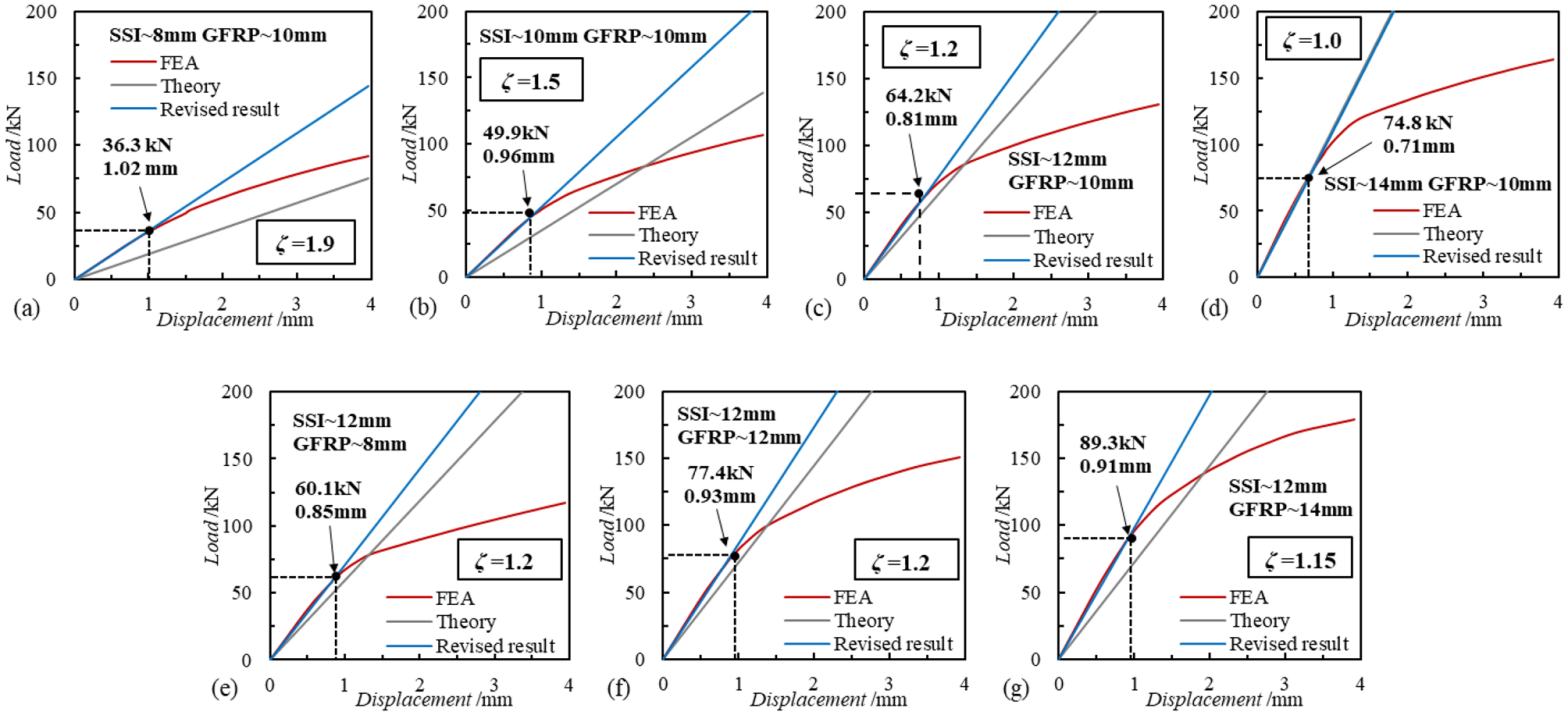
Theoretical analysis with different diameters of SSI and GFRP connectors.

Furthermore, Fig. 22 demonstrates the theoretical analysis results for different diameters of the connectors. In order to facilitate the analysis, the control variable method is used to analyze the effect of the variation of different connector diameters on the theoretical analysis of SSI-GFRP shear performance. Observing the four figures (a)–(b), when the diameter of the GFRP connectors is 8 mm, the difference between the theoretical analysis and the results of the finite elements gradually decreases as the diameter of the SSI connectors increases from 8 to 14 mm. It can be observed that the value of the theoretical correction factor also decreases from 1.9 to 1.0, and the value of the load at the end of the elastic section gradually increases. When the diameter of the SSI connector is kept as 12 mm, with the increase of the diameter of the GFRP connector, the difference between the theoretical analysis results and the finite element results is basically unchanged, and the theoretical correction coefficient *ζ* is kept in the range of 1.15–1.2. This phenomenon indicates that the change in the diameter of the GFRP connectors does not cause changes in the theoretical analysis error.

Combining the results of the theoretical analyses of the two different parameter variations, it can be seen that there are limitations in the application of the classical theory, which differs from the elastic section of the finite element analysis, and it is not possible to predict the shear performance of the SSI-GFRP connectors more accurately. When the diameter of the connectors is kept constant, the number ratio of SSI and GFRP connectors has less influence on the theoretical correction factor. When the number ratio of the connectors is kept constant, the change in the diameter of the SSI connectors has a greater effect on the theoretical correction factor, which affects the accuracy of the results of the classical theoretical analysis, and the change in the diameter of the GFRP connectors has a smaller effect on the theoretical correction factor.

## Conclusion

This study conducted a large-scale push-out test on a PCSP with intersecting SSI and GFRP connectors, observing and analyzing the failure process, failure mode, and load displacement variation of the wall panel. In addition, the effects of parameters such as the ratio of the number of connectors, the diameter of the connectors, and the strength of the concrete of the external wall on the load–displacement relationship of the SSI-GFRP connectors were also investigated using numerical analysis and verified by theoretical methods, so as to gain further insights into the comprehensive shear performance of SSI-GFRP cross-distributed connectors. In summary, the main following conclusions are drawn as follows:

1. As the horizontal thrust increases, there are different phases in the load–displacement curve of the external panel. There are elastic (0–30 kN) and elastic–plastic (30–115 kN) phases in the shear operating properties of SSI-GFRP connectors. Upon reaching a load of 115 kN, the concrete at the end of the external wall experienced cracking and localized crushing, prompting the termination of the test.
2. A proportional numerical analysis model of PCSP was created using Abaqus finite element software, and the numerical analysis results were generally consistent with the development patterns observed in the experimental load displacement curves, with a maximum difference of no more than 20%. Furthermore, the tensile damage contours of external concrete walls indicated noticeable damage at the locations where the connectors were installed, mirroring the experimental findings, which verifies the effectiveness of the numerical analysis model.
3. According to the numerical analysis results, the number ratio of SSI to GFRP connectors and the diameter of the connectors have a significant effect on the combined shear performance of SSI-GFRP connectors, and the final load of the external concrete wall increases with the increase of the above two parameters. The concrete strength of the PCSP has almost no effect on the combined shear performance of SSI-GFRP connectors, but with the increase of the concrete strength, the wall panels' plastic damage point and damage degree gradually decrease.
4. The classical theory for calculating the deformation of the connectors has some limitations in its application, and a theoretical correction factor (*ζ*) is given to improve the accuracy of the theoretical results. The number ratio of different connectors has little effect on the theoretical correction coefficient, while the change in the diameter of SSI connectors has a greater effect on the theoretical correction coefficient, which needs to be taken into account in the application.

## Data Availability

All data generated or analyzed during this study are included in this article.
